# 5-(Benzofuran-2-yl)-3-(2-chloro-4-fluorobenzyl)-1,3,4-oxadiazol-2(3H)-one (GM-90663) Alleviates Dravet Syndrome via Inhibiting Monoamine Oxidase Activity

**DOI:** 10.3390/molecules31091511

**Published:** 2026-05-01

**Authors:** Kyu-Seok Hwang, Se Hwan Ahn, Yuji Son, Seong Soon Kim, Dae-Seop Shin, Jung Yoon Yang, Chong Hak Chae, Michiko Nakamura, Il-Sung Jang, Gahyeon Kim, Dong Gun Kim, Pyeongkeun Kim, Yerim Heo, Sunjae Bae, Hohjai Lee, Jin Hee Ahn, Myung Ae Bae

**Affiliations:** 1 Therapeutics & Biotechnology Division, Korea Research Institute of Chemical Technology, Daejeon 34114, Republic of Korea; kshwang@krict.re.kr (K.-S.H.); yjson423@gmail.com (Y.S.); kimss@krict.re.kr (S.S.K.); dsshin@krict.re.kr (D.-S.S.); yjy1608@krict.re.kr (J.Y.Y.); chchae@krict.re.kr (C.H.C.); kimgh@krict.re.kr (G.K.); 2 Department of Chemistry, Gwangju Institute of Science and Technology, Gwangju 61005, Republic of Korea; ansehwan@gm.gist.ac.kr (S.H.A.); lagnaloker0@gm.gist.ac.kr (D.G.K.); kpk0911@gm.gist.ac.kr (P.K.); yrheo018@gm.gist.ac.kr (Y.H.); bsjkr@gm.gist.ac.kr (S.B.); 3 Department of Pharmacology, School of Dentistry, Kyungpook National University, Daegu 41940, Republic of Korea; michiko21a@gmail.com (M.N.); jis7619@knu.ac.kr (I.-S.J.); 4 Brain Science & Engineering Institute, Kyungpook National University, Daegu 41940, Republic of Korea; 5 Department of Medical Chemistry and Pharmacology, University of Science & Technology, Daejeon 34113, Republic of Korea; 6 JD Bioscience Inc., 208 Cheomdan-dwagiro, Buk-gu, Gwangju 61011, Republic of Korea

**Keywords:** Dravet syndrome, treatment, monoamine oxidase, SCN1A, zebrafish

## Abstract

Dravet syndrome (DS) is a severe, catastrophic childhood epilepsy predominantly caused by loss-of-function mutations in the *SCN1A* gene, which encodes the voltage-gated sodium channel *Na_v_*1.1. In this study, we evaluated the therapeutic potential of 5-(Benzofuran-2-yl)-3-(2-chloro-4-fluorobenzyl)-1,3,4-oxadiazol-2(3H)-one (GM-90663), a novel small molecule designed to address the complex pathophysiology of DS. Using *scn1lab* knockout (KO) zebrafish larvae—a robust vertebrate model for DS—we demonstrated that GM-90663 significantly alleviates seizure-like behavioral movements and rescues deficit in cognitive-like functions. Whole-cell patch-clamp recordings in hippocampal slices revealed that GM-90663 modulates voltage-gated Na^+^ channel kinetics; specifically, it suppresses slow ramp-induced currents, thereby effectively attenuating neuronal hyperexcitability. Furthermore, neurochemical profiling indicated that GM-90663 treatment leads to a marked increase in endogenous serotonin (5-HT) levels in both wild-type and KO larvae. Molecular docking simulations and subsequent in vitro enzymatic assays confirmed that this elevation in serotonin is mediated through the potent inhibition of monoamine oxidase (MAO) activity. Collectively, our findings suggest that GM-90663 exerts its anti-seizure effects through a synergistic dual mechanism—stabilizing sodium channel conductance and elevating serotonergic activity—positioning it as a promising multi-target candidate for the treatment of DS.

## 1. Introduction

Epilepsy is a disorder of the central nervous system (CNS) in which the pathological state is exerted through repeated episodes of excessive neural activity, known as seizures. Through genome-wide association sequencing (GWAS) or exome sequencing studies, epilepsy-associated sequence variants in hundreds of genes have been identified [1]. Dravet syndrome (DS) is caused by a monogenic mutation in *SCN1A*, which encodes a voltage-gated sodium channel (Nav1.1). It is a clinically relevant epilepsy gene with thousands of genetic variants reported in different phenotypes. The most frequent phenotype is febrile seizures plus (FS+), and afebrile generalized tonic–clonic seizures [2]. Most infants with DS exhibit normal development before experiencing an increase in the frequency of febrile and afebrile seizures during the first year. Eventually, symptoms progress to severe recurrent seizures, cognitive dysfunction, psychomotor impairment, and intellectual disability [3,4].

Although anti-seizure drugs (ASDs) such as valproate (VPA), clobazam (CLB), and stiripentol (STP) are used to alleviate seizures in DS, seizures remain poorly controlled [5]. Despite the availability of >30 ASDs with broad categories of mechanisms, new ASDs with different mechanisms are needed to effectively reduce seizures while improving other comorbidities [6]. DS is primarily characterized by the loss-of-function of the *SCN1A* gene, which predominantly affects GABAergic inhibitory interneurons, leading to a global imbalance between excitation and inhibition in the brain. While current therapeutic strategies often focus on enhancing GABAergic neurotransmission, many patients remain refractory to these treatments. In the last decade, three ASDs were specifically available as therapeutic agents in DS: STP, cannabidiol (CBD), and fenfluramine (FFA). Although the most commonly used treatments are VPA and CLB in DS, these ASDs were escalated in efficacy as adjunctive therapy [5]. STP received approval as a first therapy for the treatment of seizures associated with DS in 2017 in the EU and 2018 in the USA. STP is likely to have several mechanisms linked to anti-seizure properties, such as a positive allosteric modulator of GABAA receptors [7,8]. CBD was approved as an adjunctive treatment for seizures in DS and Lennox–Gastaut syndrome (LGS) in 2018 in the USA and in 2019 in the EU. Unlike other ASDs, CBD has distinct mechanisms in that it modulates intracellular calcium and inhibits adenosine cellular uptake [9]. FFA, approved in 2020 in the EU and USA, is an add-on therapeutic agent for the treatment of seizures in patients with DS. Originally, FFA was used as a therapy for obesity until it was withdrawn because of increased cases of cardiac problems [5,10]. FFA acts variously on the serotonergic system compared with other ASDs: increasing serotonin (5-hydroxytryptamine, 5-HT) release, inhibiting the serotonin transporter, and agonist of serotonin receptors [11]. In addition, FFA functions as a positive modulator of the sigma-1 receptor that could improve cognitive functions [12]. Although the pharmacological profile of fenfluramine (FFA) has been a subject of ongoing debate, recent studies have shifted the consensus towards its role as a positive modulator of the sigma-1 receptor. While early zebrafish reports hypothesized a complex interaction with sigma-1 receptor [13], current evidence supports that FFA’s agonistic activity at this receptor, alongside its serotonergic effects, contributes to its potent anti-seizure properties in SCN1A-deficient models [12].

Several studies to generate and characterize the animal models of DS for drug screening have been reported. Zebrafish (*Danio rerio*) has emerged as a promising in vivo model for biomedical research in the nervous system [14,15]. In addition, they are an attractive model for studying genetic diseases because approximately 70% of coding genes are conserved between humans and zebrafish [16]. Since the mutation of the *SCN1A* ortholog in zebrafish was reported, several studies have made efforts to find a small molecule for the treatment of DS [17,18,19,20,21,22]. The first reported *scn1lab* mutant in zebrafish, *didy^s552^*, was generated by chemical mutagenesis and identified as a mutant with a point mutation [23,24]. FFA is selected as an anti-seizure compound by large-scale screening in a zebrafish model of DS [18]. In addition, the zebrafish model of DS revealed that the anti-seizure effect of FFA not only acts as a 5-HT1D- and 5-HT2C-agonist, but likely also functions to block sigma-1 receptor [13]. These studies demonstrated that the serotonergic system can be a good target to restore DS-associated seizures.

Previously, we generated a new *scn1lab* knockout (KO) zebrafish using the CRISPR/Cas9 method to find a novel therapeutic agent for the treatment of DS [25], and identified compound **20e** (3-(2-Chloro-4-fluorobenzyl)-5-(2-(trifluoromethyl)pyridin-4-yl)-1,3,4-oxadiazol-2(3H)-one), which demonstrated potent anti-seizure efficacy by upregulating TPH2 (tryptophan hydroxylase 2) expression. During the optimization, we synthesized diverse molecules. Among the synthesized compounds, we found 5-(Benzofuran-2-yl)-3-(2-chloro-4-fluorobenzyl)-1,3,4-oxadiazol-2(3H)-one (GM-90663), which alleviated Dravet syndrome with MOA inhibition as well as modulation of voltage-gated Na^+^ channel. This situation prompted us report the synthesis, molecular docking and biological evaluation of new candidate for DS.

## 2. Materials & Methods

### 2.1. Materials

Commercially available reagents and solvents were utilized directly unless otherwise specified. The yields provided correspond to isolated products purified via crystallization or silica gel column chromatography. Proton nuclear magnetic resonance (^1^H NMR) data were acquired utilizing a JEOL JNM-ECS400 instrument (JEOL Ltd., Tokyo, Japan) operating at 400 MHz. Spectra were referenced internally to tetramethylsilane (TMS), with chemical shifts (δ) reported in ppm using CDCl_3_ as the deuterated solvent. Signal splitting patterns are designated as follows: s, singlet; d, doublet; t, triplet; q, quartet; dd, doublet of doublets; ddd, doublet of doublet of doublets; qd, quartet of doublets; dt, doublet of triplets; m, multiplet. For HPLC characterization, a Waters Agilent platform coupled with a PDA detector and a Waters SB-C18 analytical column (1.8 μm, 2.1 × 50 mm) was employed. Elution was carried out at a flow rate of 0.5 mL/min, employing a gradient of highly purified water containing 0.1% trifluoroacetic acid (Eluent A) and chromatographic-grade acetonitrile (Eluent B).

### 2.2. Procedure for the Synthesis of Compound GM-90663

Step 1. To a solution of ethyl benzofuran-2-carboxylate, **1** (1.25 g, 6.572 mmol) in EtOH (100 mL) was added hydrazine hydrate (2.303 g, 46.004 mmol, 7.0 equiv). Following 6 h of reflux, the mixture was concentrated in vacuo. The resulting residue was diluted with H_2_O and extracted with EtOAc (3 × 100 mL). The combined organic layers were dried over Na_2_SO_4_ and evaporated to afford benzofuran-2-carbohydrazide, **2** (880 mg, 4.995 mmol, 76%). ^1^H NMR (400 MHz, Chloroform-*d*) δ 7.80 (s, 1H), 7.69 (dd, J = 7.8, 1.3, 0.7 Hz, 1H), 7.54–7.48 (m, 2H), 7.43 (ddd, J = 8.5, 7.1, 1.3 Hz, 1H), 7.31 (ddd, J = 8.1, 7.1, 1.1 Hz, 1H), 4.10 (s, 2H).

Step 2. To a stirred solution of Benzofuran-2-carbohydrazide, **2** (880 mg, 4.995 mmol) and Et_3_N (2.089 mL, 14.985 mmol, 3.0 equiv) in THF (10 mL) at 0 °C, a solution of triphosgene (1.482 g, 4.995 mmol, 1.0 equiv) in THF was added dropwise. The reaction mixture was stirred at 0 °C for 1 h, then gradually warmed to room temperature and refluxed for 6 h. After cooling, the mixture was concentrated under reduced pressure. Purification of the resulting residue by silica gel column chromatography afforded 5-(benzofuran-2-yl)-1,3,4-oxadiazol-2(3H)-one, **3** (780 mg, 3.858 mmol, 77%). ^1^H NMR (400 MHz, DMSO-*d_6_*) δ 12.86 (s, 1H), 7.78 (dd, J = 7.8, 1.2 Hz, 1H), 7.73 (d, J = 8.3 Hz, 1H), 7.60 (d, J = 1.0 Hz, 1H), 7.48 (ddd, J = 8.5, 7.2, 1.4 Hz, 1H), 7.37 (dd, J = 7.9, 7.0 Hz, 1H).

Step 3. Under a nitrogen atmosphere, 5-(benzofuran-2-yl)-1,3,4-oxadiazol-2(3H)-one, **3** (150 mg, 0.742 mmol) and 2-chloro-4-fluorobenzyl bromide (164.65 mg, 0.742 mmol, 1.0 equiv) were dissolved in anhydrous DMF (2 mL). A suspension of NaH (60% in mineral oil, 17.81 mg, 0.742 mmol, 1.0 equiv) in DMF was added dropwise. After stirring at 60 °C for 4 h, the mixture was concentrated. The crude was partitioned between H_2_O (20 mL) and EtOAc (3 × 50 mL). The organic extracts were washed with brine and chromatographed on silica gel to yield 5-(benzofuran-2-yl)-3-(2-chloro-4-fluorobenzyl)-1,3,4-oxadiazol-2(3H)-one, **GM-90663** (147.7 mg, 0.430 mmol, 58%). ^1^H NMR (400 MHz, Chloroform-*d*) δ 7.70–7.63 (m, 1H), 7.56 (d, J = 8.2 Hz, 1H), 7.47–7.38 (m, 2H), 7.36–7.28 (m, 2H), 7.26 (s, 1H), 7.18 (dd, J = 8.4, 2.6 Hz, 1H), 7.02 (td, J = 8.3, 2.6 Hz, 1H), 5.10 (s, 2H). LC-MS (*m*/*z*): 345.1 [M + H]^+^; HPLC purity 98.69%.

### 2.3. Maintenance of Zebrafish

Zebrafish embryos and larvae were reared under standard laboratory conditions as established by The Zebrafish Book [26]. Briefly, fertilized eggs were collected after natural spawning and selected using a stereomicroscope to remove unfertilized eggs. Embryos were kept in embryonic medium (60 μg/L of sea salt; Sigma-Aldrich, St. Louis, MO, USA, cat# S9883) at 28 °C with a 14:10 h light/dark photoperiod. Embryonic medium was replaced daily until 6 days post-fertilization (dpf). Experiments involving zebrafish were performed in accordance with the NIH Guide for the Care and Use of Laboratory Animals (no. 8023, revised in 1996), and this study was approved by the Animal Care and Use Committee of the Korea Research Institute of Chemical Technology (2021-7B-04-02).

### 2.4. Behavioral Analysis in scn1lab Knockout (KO) Zebrafish Larvae

Larvae were obtained from the breeding of *scn1lab^kri111/+^* (AB strain) adults [25]. We utilized homozygous KO individuals, identified by their characteristic hyper-pigmentation, alongside age-matched wild-type siblings. To ensure data integrity, only larvae with normal morphological development were randomly assigned to experimental groups. Regarding the experimental timeline, all larvae were allowed to acclimatize to the testing environment during the morning hours. Behavioral assessments were consistently conducted in the afternoon to ensure stable physiological conditions and to minimize variability related to the animals’ circadian rhythms. At 6 days post-fertilization (dpf), individual larvae were transferred to 96-well plates and dark-acclimated for 30 min within a tracking chamber. GM-90663 was prepared as a 2 mM stock in dimethyl sulfoxide (DMSO) and subsequently diluted in embryo medium to final concentrations (0.5–2 μM). The final concentration of DMSO in all treatment and vehicle control groups was 0.1% (*v*/*v*), which has been reported to have no significant effect on zebrafish larval behavior or development. Larval activity was monitored for 30 min using the DanioVision system (Noldus, Wageningen, the Netherlands) equipped with EthoVision XT 15 software (Noldus). Seizure-like behaviors were categorized according to established criteria [25,27]. To evaluate color preference, 6-dpf larvae were pre-treated with either DMSO or GM-90663 in 6-well plates for 4 h. Following treatment, groups of 20 larvae were introduced into a dual-channel chamber (blue and yellow) containing 10 mL of embryo medium [28]. A digital camera (HDR-CX130, Sony, Tokyo, Japan) recorded the distribution of larvae for 30 min. To quantify positional data, static images were captured from the video recordings at 2 min intervals. Preference scores for each color were determined by calculating the mean occupancy percentage of larvae within each respective channel over the recording period.

### 2.5. Electrophysiology

Neurons were obtained from hippocampal slices prepared from Sprague–Dawley rats (postnatal day 12–17, both sexes). Animals were anesthetized with ketamine (50 mg/kg, intraperitoneal) and subsequently decapitated. The brain was rapidly removed and sectioned transversely into 400 µm thick slices using a vibrating microslicer (Campden Instruments, Leicester, UK). To preserve tissue viability, slices containing the hippocampus were kept in an incubation medium (in mM; 124 NaCl, 3 KCl, 1.5 KH_2_PO_4_, 24 NaHCO_3_, 2 CaCl_2_, 1.3 MgSO_4,_ and 10 glucose) saturated with 95% O_2_ and 5% CO_2_ at room temperature (22–25 °C) for at least 1 h before the mechanical dissociation. For acute dissociation, individual slices were transferred into a 35 mm culture dish (Primaria 3801; Becton Dickinson, Rutherford, NJ, USA), which contained the standard external solution (in mM; 150 NaCl, 3 KCl, 2 CaCl_2_, 1 MgCl_2_, 10 glucose and 10 HEPES, a pH of 7.4 with Tris-base). The CA3 region of the hippocampus was visually identified under a stereomicroscope (SMZ-1; Nikon, Tokyo, Japan), and neurons were mechanically isolated as previously described [29,30]. Briefly, mechanical dissociation was performed using a vibration device (SI-10; K.T. Labs., Fukuoka, Japan) and a fire-polished glass pipette oscillating at approximately 50–60 Hz (0.3–0.5 mm) over the hippocampal CA3 region. Following this procedure, the slices were removed from the dish, and the acutely dissociated neurons were allowed to settle onto the bottom surface for approximately 15 min before further experimentation.

Whole-cell voltage-clamp recordings were performed using a patch-clamp amplifier (MultiClamp 700B; Molecular Devices, San Jose, CA, USA) following standard procedures [31]. Unless otherwise specified, cells were held at −100 mV. Patch electrodes were fabricated from borosilicate glass capillaries (Narishige, Tokyo, Japan) using a programmable puller (Sutter Instrument, Novato, CA, USA) and had a resistance of 1.0–1.5 MΩ when filled with the internal solution. The pipette solution contained (in mM): 140 CsF, 10 CsCl, 2 EGTA, 2 Na_2_-ATP, and 10 HEPES, adjusted to pH 7.2 with Tris base. Membrane currents were low-pass fil-tered at 3 kHz, digitized at 10 kHz, and acquired using pCLAMP 10.7 software (Molecular Devices). Capacitive transients and linear leak components were corrected using a P/4 subtraction protocol. Series (access) resistance was continuously monitored by applying brief hyperpolarizing voltage steps (10 mV, 30 ms), and recordings were excluded if the resistance varied by more than 10% during the experiment. To isolate voltage-gated Na^+^ current (I_Na_), recordings were conducted in a K^+^-free external solution (in mM; 130 NaCl, 20 tetraethylammonium-Cl, 3 CsCl, 2 CaCl_2_, 1 MgCl_2_, 10 glucose, and 10 HEPES, and was adjusted to pH 7.4 with Tris-base). In addition, the bath solution was supplemented with 10 μM SR95531, 20 μM CNQX, 50 μM DL-APV, 100 μM Cd^2+^ to suppress GABA_A_ receptors, ionotropic glutamate receptors, and voltage-gated Ca^2+^ channels. Depolarizing voltage steps were delivered at 10 s intervals to allow complete recovery from Na^+^ channel inactivation, unless stated otherwise. Pharmacological agents were applied using the “Y-tube system” for rapid solution exchange, as previously described [32]. All experiments were performed at room temperature (22–25 °C).

Peak I_Na_ amplitude was determined as the difference between baseline and maximal inward current (pCLAMP 10.7). In a subset of experiments, the amplitude of TTX-R I_Na_, was transformed into conductance (G) using the following equation: G = I/(V − E_Na_), where E_Na_ is the Na^+^ equilibrium potential (+87.7 mV) calculated by the Nernst equation. The voltage-activation and voltage-inactivation relationships of TTX-R Na^+^ channels were fitted to the following Boltzmann equations, respectively; G/G_max_ = 1/{1 + exp[(V_50,activation_ − V)/*k*]} and I/I_max_ = 1 − 1/{1 + exp[(V_50,inactivation_ − V)/*k*]}, where G_max_ is the maximum conductance, I_max_ is the maximum current amplitude, V_50,activation_ is the half-maximum potential for activation, V_50,inactivation_ is the half-maximum potential for fast inactivation, and *k* is the slope factor. The kinetic data for the recovery from inactivation were fitted to the following equation: R(*t*) = A_0_ + A_fast_ × [1 − exp(–*t*/τ_fast_)] + A_intermediate_ × [1 − exp(–*t*/τ_intermediate_)] + A_slow_ × [1 − exp(–*t*/τ_slow_)], respectively, where R(*t*) is the amplitude ratio of I_Na_ at time *t*, and A_fast_, A_intermediate_, and A_slow_ are the amplitude fractions of τ_fast_, τ_intermediate_, and τ_slow_, respectively.

### 2.6. Quantitative Real-Time Polymerase Chain Reaction (qRT-PCR)

Following compound exposure, total RNA was extracted from 20-pooled larvae (WT or KO) using TRIzol (Invitrogen, cat# 15596026) reagent, following the standard manufacturer’s instructions. qRT-PCR was performed using 1-Step qRT-PCR kit (Thermo Scientific, Waltham, MA, USA, cat# AB-4106/A) with 7500/7500 Fast Real-Time PCR system (Applied Biosystems, Waltham, MA, USA,) under the manufacturer’s specified thermal conditions. Relative mRNA expression levels were determined via the 2^−ΔΔct^ method, with *β-actin* serving as the internal reference gene for normalization. The specific primer sequences utilized are detailed in Table 1.

### 2.7. Immunohistochemistry

Immunohistochemistry for phosphorylated Extracellular Signal-Regulated Kinases (pERK) was conducted as previously described [33]. To preserve transient phosphorylation states, larvae were placed in 6-well plates with cell strainers (SPL, cat# 93100) to facilitate rapid immersion in fixative. Following a 4 h chemical treatment, samples were immediately fixed in a solution of 4% paraformaldehyde and 4% sucrose in phosphate-buffered saline (PBS) overnight at 4 °C. After fixation, tissues were rinsed with PBS containing 0.25% Triton X-100 (PBTx), and larval brains were carefully micro-dissected to optimize antibody accessibility. The tissues were then permeabilized with 1 mg/mL collagenase (Sigma-Aldrich, cat# C9891) in PBS for 1 h, followed by overnight blocking in PBTx supplemented with 2% normal goat serum and 2% DMSO at 4 °C. Brain tissues were incubated overnight with a primary rabbit anti-pERK antibody (Cell signaling Technology, Danvers, MA, USA, cat# 4370) and subsequently with a secondary antibody (Invitrogen, cat# A-11034). After final washes, the stained tissues were embedded in 1% low-melting-point agarose and visualized using a K1-Fluo confocal fluorescence microscopy (Nanoscope Systems, Daejeon, Republic of Korea).

### 2.8. LC-MS/MS

Comprehensive neurochemical analysis was conducted in zebrafish larvae (30-pooled) following exposure to GM-90663 in wild-type or KO. This analysis utilized an ultra-performance liquid chromatography (ACQUITY UPLC System, Waters Corporation, Milford, MA, USA) coupled with a Xevo TQ-S triple quadrupole mass spectrometer (LC-MS/MS, Waters Corporation), as per a previously reported method [34]. Briefly, the zebrafish larvae were washed three times in ice-cold 0.1 M phosphate-buffered saline (PBS, pH 7.4, Corning, Corning, NY, USA), snap-frozen in liquid nitrogen. The samples were homogenized by ultrasonication in 100 μL of distilled water, extracted with an equal volume of methanol, vortexed, and centrifuged at 15,000 RPM at 4 °C for 20 min. The supernatant was then transferred to an LC-vial for the quantitative analysis of 31 neurochemicals using LC-MS/MS.

### 2.9. Molecular Modeling

The Schrodinger Suite was used for the molecular modeling study [35]. The protein structural files of human MAO-A and MAO-B were obtained from the Protein Data Bank (PDB codes: 2Z5X and 2V5Z, respectively). Preparation of the protein models, including bond order assignment, addition of hydrogen atoms, and correction of missing loops and side chains, was performed by using the Protein Preparation Wizard of Schrodinger suite. The protein-ligand complexes were relaxed with Impref using the OPLS4 force field. Since the experimental structure of zebrafish MAO (zMAO) is not published yet, the AlphaFold prediction model (AF-Q6NSN2-F1; https://alphafold.ebi.ac.uk/entry/Q6NSN2, accessed on 1 Auguest 2025) was employed as the protein model. Docking simulations were performed using the Glide docking program with the standard SP flexible ligand docking protocol and default settings. The ligand structures were docked into the energy grids of MAO proteins. The receptor grids for each MAO protein were generated using the Receptor Grid Generation module. The ligand binding sites were defined by the centroid of the crystal ligand with a cubit grid size of 22 Å. Robustness of protein structure and docking protocol was verified via reproduction of crystal ligand poses with a good root mean square displacement of 0.5 Å. The best-energy pose, having the lowest Glide score, was selected for the subsequent binding pose analysis and molecular dynamics simulation. The Desmond software with default settings was used in the classical MD simulations of the complexes for the interaction analysis. The structures of the best-scoring poses of protein-ligand complexes in each MAO protein were solvated in the SPC water model and the OPLS4 forcefield using orthorhombic periodic boundary conditions, with a 10 Å buffer distance and neutralizing Na^+^ or Cl^−^ ions. Production simulations were run for 100 ns with constant temperature and pressure of 300 K and 1.01325 bar. After 100 ns simulations of each complex, the lowest-energy conformations were obtained from the last 10 ns trajectories. The sampling interval during the simulation was set to 100 ps, and the analysis of simulation results was performed using the SID and SEA programs in Maestro.

### 2.10. Monoamine Oxidase (MAO) Assay in Zebrafish Larvae

Assay of MAO activity was performed using a commercial kit (abcam, Cambridge, UK, cat# ab241031). Homogenates were prepared from 20-pooled larvae after exposure to GM-90663 in wild-type or KO. MAO activity was measured using a microplate reader (TECAN, Männedorf, Switzerland, M100PRO) as a fluorescence intensity (Ex/Em = 535/587 nm) and calculated according to the manufacturer’s protocol.

### 2.11. Statistical Analysis

Data are presented as mean ± standard error of mean (SEM). Statistical comparisons were performed via the non-parametric Mann–Whitney test using GraphPad Prism 9.4.0 and Microsoft Excel 2016. Electrophysiological data are presented as mean ± SEM, with normalization to control values where appropriate. Statistical comparisons were performed using paired two-tailed Student’s t-tests on absolute current amplitudes. A *p*-value of less than 0.05 was adopted as the criterion for statistical significance (* *p* < 0.05, ** *p* < 0.01, and *** *p* < 0.001).

## 3. Results

### 3.1. Synthesis of GM-90663

**Scheme 1:**
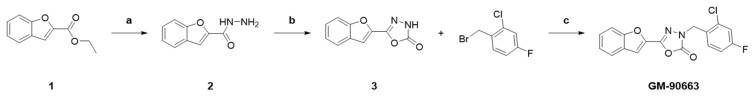
Synthesis of compound GM-90663. Reagents and conditions: (a) NH_2_OH·HCl, TEA, EtOH, reflux, 76%; (b) Triphosgene, TEA, THF, reflux, 77%; (c) NaH, DMF, 60 °C, 58%.

As illustrated in Scheme 1, **GM-90663** was synthesized from ethyl benzofuran-2-carboxylate via a multi-step procedure. Initially, ethyl benzofuran-2-carboxylate (**1**) was transformed into benzofuran-2-carbohydrazide (**2**) via hydrazinolysis with hydrazine hydrate. Intermediate 2 was then cyclized using triphosgene in the presence of TEA to yield 5-(benzofuran-2-yl)-1,3,4-oxadiazol-2(3H)-one (**3**). Subsequently, intermediate **3** underwent N-alkylation with 2-chloro-4-fluorobenzyl bromide in the presence of sodium hydride to afford **GM-90663**.

### 3.2. GM-90663 Treatment Exhibits Anti-Seizure Effects in scn1lab Knockout (KO) Zebrafish Larvae

**Figure 1:**
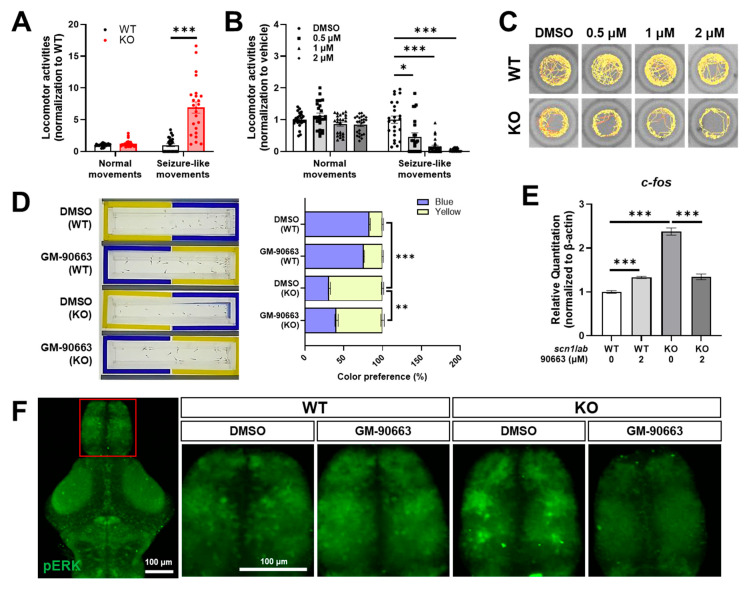
Anti-seizure effects of GM-90663 in *scn1lab* KO zebrafish larvae. (**A**) Measurement of normal and seizure-like movements across WT and *scn1lab* KO larvae (*n* = 8). (**B**) Measurement of normal movements and seizure-like movements across WT and *scn1lab* KO larvae following GM-90663 exposure (*n* = 8). (**C**) Visual representation of larval movement traces over 30 min period (*n* = 8). (**D**) Color preference outcomes for WT and *scn1lab* KO larvae following GM-90663 exposure (*n* = 20). (**E**) pERK immunostaining results and (**F**) quantitative *c-fos* mRNA levels measured in WT and *scn1lab* KO larvae exposed to GM-90663, respectively (*n* = 10). *; *p* < 0.05, **; *p* < 0.01, and ***; *p* < 0.001.

We tried to identify a potential therapeutic agent for the treatment of Dravet syndrome (DS) using a *scn1lab* KO zebrafish [25]. In this study, 5-(benzofuran-2-yl)-3-(2-chloro-4-fluorobenzyl)-1,3,4-oxadiazol-2(3H)-one (hereafter referred to as GM-90663), which consists of the benzofuran, 1,3,4-oxadiazolone, and chlorofluorobenzyl moiety, was identified. (Scheme 1). A dose-dependent attenuation of seizure-like movements was observed in *scn1lab* knockout larvae following the administration of GM-90663 (Figure 1A–C). However, wild-type or heterozygous sibling larvae did not exhibit behavioral changes with GM-90663 treatment.

It is well-documented that patients suffering from Dravet syndrome (DS) and other forms of epilepsy often experience cognitive deficits secondary to chronic seizure activity [3,36]. To assess similar cognitive parameters in our in vivo model, we utilized a color preference assay, taking advantage of the natural color-discriminating visual abilities of zebrafish [37]. In contrast to wild-type and heterozygous counterparts, which exhibited a clear preference for blue environments over yellow, the *scn1lab* mutant larvae displayed an inverted color choice. Following the administration of GM-90663, this abnormal color bias in the knockout models was substantially reversed, shifting their preference back toward blue (Figure 1D).

The immediate-early gene *c-fos* is a widely accepted marker of neuronal activity, used to map brain regions responsive to various stimuli [38]. PTZ-induced seizures are known to increase *c-fos* expression across various regions of the zebrafish brain [39]. In this study, while *c-fos* expression was significantly upregulated in *scn1lab* KO larvae, GM-90663 treatment restored these expression levels to those observed in wild-type (WT) larvae (Figure 1E). pERK is an endogenous sensor to determine neural activity. Pentylenetetrazole (PTZ) is a GABA_A_ receptor antagonist that induces seizure-like behavior and widespread activation of pERK level in zebrafish [27,40]. To test whether GM-90663 decreases neural activity in *scn1lab* KO zebrafish larvae, we performed immunohistochemistry for pERK. The level of pERK in *scn1lab* KO larvae was enriched in the telencephalon region, whereas GM-90663 treatment reduced it (Figure 1F). These results demonstrate that GM-90663 effectively restored seizure-like behaviors and neural activities in *scn1lab* KO zebrafish larvae.

### 3.3. Effects of GM-90663 on Voltage-Gated Na^+^ Channels in Hippocampal CA3 Neurons

**Figure 2:**
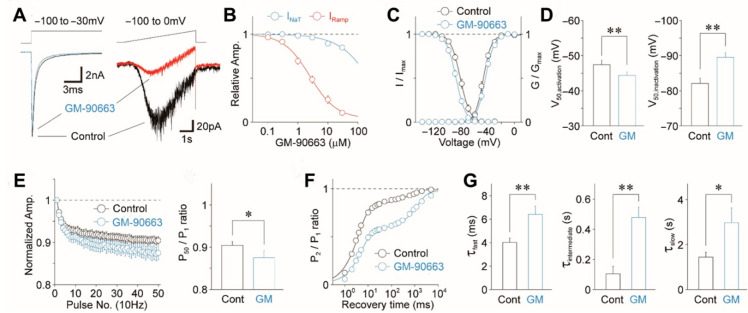
Effect of GM-90663 on voltage-gated Na^+^ channels in acutely isolated rat hippocampal CA3 neurons. (**A**) Typical traces of I_Na_ (**left**) and I_Ramp_ (**right**) in the absence and presence of 10 μM GM-90663. The I_Na_ (**left**) was elicited by electrical stimulation from a holding potential of −100 mV to −30 mV (100 ms duration) at every 5 s. The I_Ramp_ was elicited by slow voltage-ramp stimuli (−100 mV to −20 mV, 5 s duration; 16 mV/s) at every 20 s. (**B**) Concentration–response relationships of GM-90663 for the I_Na_ (cyan circles) and I_Ramp_ (red circles). A continuous lines were fitted using a least squares method. Points and error bars represent the mean and SEM from 6 neurons. (**C**) Voltage-activation and voltage-inactivation relationships of Na^+^ channels in the absence (black circles) and presence (cyan circles) of 10 μM GM-90663. Continuous lines were fitted using the Boltzmann function. Points and error bars represent the mean and SEM from 6 neurons for activation and 7 neurons inactivation experiments. (**D**) GM-90663-induced changes in the midpoint voltages for activation (V_50, activation_, **left**) and inactivation (V_50, inactivation_, **right**) of voltage-gated Na^+^ channels. Columns and error bars represent the mean and SEM from 6 neurons for activation and 7 neurons inactivation experiments. **; *p* < 0.01. (**E**) Time course of the normalized peak amplitude of the I_Na_ during a train of 50 pulses (10 Hz) in the absence (black circles) and presence (cyan circles) of GM-90663 (**left**). Points and error bars represent the mean and SEM from 6 neurons. GM-90663-induced changes in the P_50_/P_1_ ratio of the I_Na_ (**right**). Columns and error bars represent the mean and SEM from 6 neurons. *; *p* < 0.05. (**F**) Kinetics for the recovery from inactivation of voltage-gated Na^+^ channels in the absence (open circles) and presence (cyan circles) of GM-90663. The P_2_/P_1_ ratios were plotted against the recovery time. Continuous lines were fitted using the double exponential function. Points and error bars represent the mean and SEM from 10 neurons. (**G**) GM-90663-induced changes in the fast (τ_fast_, **left**), intermediate (τ_intermediate_, **middle**), and slow (τ_slow_, **right**) time constants for the recovery from inactivation of voltage-gated Na^+^ channels. Columns and error bars represent the mean and SEM from 10 neurons. *; *p* < 0.05, **; *p* < 0.01.

Since a number of anti-epileptic drugs act on voltage-gated Na^+^ channels to decrease neuronal excitability [41], we examined whether GM-90663 affects various properties of these channels in acutely isolated CA3 neurons using the whole-cell patch-clamp technique. The voltage-gated Na^+^ current (I_Na_) was elicited by a brief depolarizing step pulse (−100 to −30 mV, every 5 s). GM-90663 slightly but significantly decreased the peak amplitude of I_Na_ to 89.3% ± 1.5% of the control (*n* = 6, *p* < 0.01). However, GM-90663 potently decreased the amplitude of slow voltage ramp-induced current (I_Ramp_) to 25.6% ± 10.8% of the control (*n* = 6, *p* < 0.01; Figure 2A). The inhibition of I_Ramp_ by GM-90663 was concentration-dependent with an IC_50_ value of 3.1 ± 0.6 μM (Figure 2B).

We also examined whether GM-90663 affects the voltage dependence of Na^+^ channels in acutely isolated CA3 neurons. The I_Na_ was elicited by 50 ms depolarizing test pulses from −100 to 0 mV in 10 mV increments in the absence and presence of GM-90663. The conductance was calculated and normalized to the respective maximal conductance, and the data were fitted to the Boltzmann function (Figure 2C). GM-90663 shifted the V_50,activation_ toward the depolarizing range (+3.0 ± 1.1 mV shift, *n* = 6, *p* < 0.01; Figure 2D).

Next, the I_Na_ was induced by test pulses (−30 mV, 50 ms duration) followed by conditioning prepulses (−140 to −30 mV in 10 mV increments, 500 ms duration). The peak amplitude of respective I_Na_ observed in the absence and presence of GM-90663 was normalized to the maximal amplitude of I_Na_, and then the normalized data were fitted to the Boltzmann function (Figure 2C). GM-90663 shifted the V_50,inactivation_ toward the hyperpolarizing range (−7.4 ± 1.5 mV shift, *n* = 6, *p* < 0.01; Figure 2D).

We further examined the effect of GM-90663 on the use-dependence of Na^+^ channels using a series of 50 depolarizing test pulses (10 Hz, 10 ms duration, up to −30 mV). When the peak amplitude of respective I_Na_ observed in the absence and presence of GM-90663 was normalized to the amplitude of the first I_Na_, GM-90663 slightly decreased the P_50_/P_1_ (0.90 ± 0.01 for the control and 0.87 ± 0.02 for the GM-90663 condition, *n* = 6, *p* < 0.05; Figure 2E).

Finally, we examined whether GM-90663 affects the recovery kinetics of voltage-gated Na^+^ channels. The I_Na_ was elicited using a two-pulse protocol, where the second test pulse (P_2_: 50 ms duration (up to −30 mV) was followed by the first conditioning pulse (P_1_; 500 ms duration, up to −30 mV) with a recovery time at −100 mV varying from 1 to 5000 ms). The P_2_/P_1_ ratios observed in the absence and presence of GM-90663 were plotted against the recovery time, and the data were fitted to a double exponential function, which yielded the kinetic parameters of the recovery from inactivation, e.g., fast (τ_fast_) and slow (τ_slow_) time constants (Figure 2F). GM-90663 significantly increased the τ_fast_ (161% ± 11%, *n* = 7, *p* < 0.01), τ_intermediate_ (465% ± 82%, *n* = 10, *p* < 0.01), and τ_slow_ (199% ± 27%, *n* = 7, *p* < 0.05) (Figure 2G).

### 3.4. Neurochemicals

**Figure 3:**
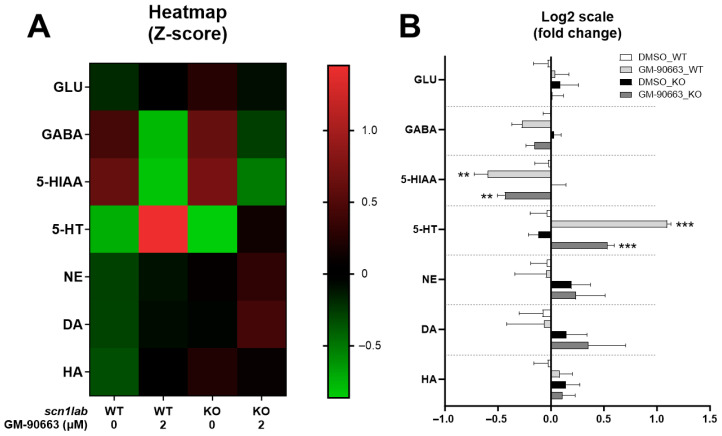
Analysis of endogenous neurochemicals in WT and in *scn1lab* KO larvae exposed to GM-90663, respectively. (**A**) Heatmap for changes in endogenous neurochemicals, with red indicating increased levels and green indicating decreased levels for each neurochemical. Normalized Z-scores were calculated based on the mean and standard deviation in experimental groups. Statistical significance to analyze differences between DMSO_WT group and experimental group was set at 0.05, 0.01 and 0.001 (** *p* ≤ 0.01 and *** *p* ≤ 0.001). (**B**) Log2 of the fold changes for endogenous neurochemicals. Each experimental group consisted of 30-pooled zebrafish larvae, and the experiment was repeated 6 times to represent the data. 5-HIAA: 5-hydroxyindoleacetic acid; 5-HT: 5-hydroxytryptamine (serotonin); DA: dopamine; GABA: gamma-aminobutyric acid; GLU: glutamic acid; HA: histamine; NE: norepinephrine.

We profiled neurochemical systems, including histaminergic, cholinergic, serotonergic, dopaminergic, and GABAergic components, in zebrafish larvae exposed to GM-90663, in both wild-type and *scn1lab* KO. There were no significant changes observed between wild-type and *scn1lab* KO zebrafish larvae. Interestingly, the results revealed that GM-90663 significantly increased 5-HT levels and decreased 5-hydroxyindoleacetic acid (5-HIAA) levels in both the wild-type and *scn1lab* KO groups compared to controls, indicating that GM-90663 potentially inhibits the metabolism of 5-HT to 5-HIAA (*p* < 0.005; Figure 3). However, GABA levels showed no significant change in either wild-type or KO zebrafish larvae exposed to GM-90663. This neurochemical profiling study underscores the impact of GM-90663 on serotonergic modulation, particularly in inhibiting the metabolic conversion of 5-HT to 5-HIAA in zebrafish larvae.

### 3.5. Differential Transcriptional Responses in scn1lab KO Larvae Following GM-90663 Exposure

**Figure 4:**
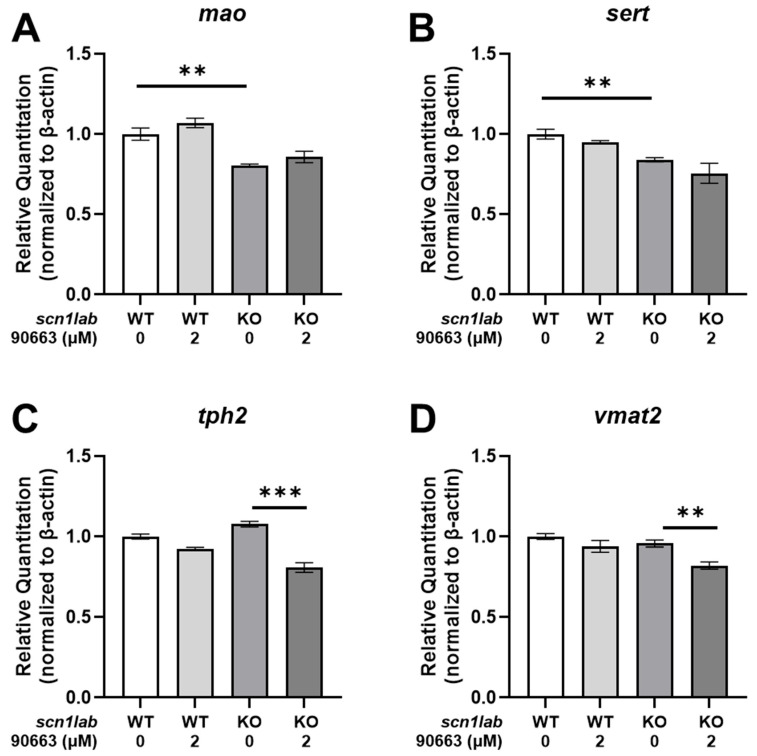
Effects of GM-90663 on the transcriptional expression of neuroactivity and serotonergic markers in zebrafish larvae. Relative mRNA expression levels of (**A**) *mao*, (**B**) *sert*, (**C**) *tph2*, and (**D**) *vmat2* were quantified using qRT-PCR in wild-type (WT) and *scn1lab* knockout (KO) larvae at 6 dpf (*n* = 10). **; *p* < 0.01 and ***; *p* < 0.001.

To determine whether the observed changes in 5-HT and 5-HIAA levels were regulated at the transcriptional level, we analyzed the expression of key genes involved in serotonin metabolism: *tph2* (synthesis), *vmat2* (vesicular transport), *sert* (reuptake), and *mao* (degradation). Interestingly, our baseline analysis revealed that *scn1lab* KO larvae exhibited significantly lower mRNA expression of *mao* (RQ = 0.8 ± 0.01, *p* < 0.01) and *sert* (RQ = 0.84 ± 0.01, *p* < 0.01) compared to wild-type (WT) larvae (Figure 4B,C). This suggests that the serotonergic system in the Dravet syndrome model is inherently compromised, potentially reflecting an incomplete compensatory response to chronic seizure activity. However, treatment with GM-90663 did not induce further alterations in the mRNA levels of any tested genes in either WT or KO groups (Figure 4A,B). These results indicate that the potent modulation of 5-HT levels by GM-90663 is not mediated through transcriptional regulation of these core serotonergic components.

Also, while GM-90663 treatment did not affect gene expression in WT larvae, it induced a specific down-regulation of *tph2* (RQ = 0.81 ± 0.03, *p* < 0.001) and *vmat2* (RQ = 0.82 ± 0.02, *p* < 0.01) exclusively in the *scn1lab* KO group (Figure 4C,D). This genotype-specific reduction likely represents a homeostatic compensatory response; the potent inhibition of MAO activity by GM-90663 markedly increases synaptic 5-HT levels, which in turn triggers a negative feedback mechanism to down-regulate 5-HT synthesis (*tph2*) and vesicular packaging (*vmat2*) to prevent excessive serotonergic signaling.

These results indicate that the potent modulation of 5-HT levels by GM-90663 is not mediated through transcriptional regulation of these core serotonergic components.

### 3.6. GM-90663 Inhibits Enzyme Activity of MAO-A and Zebrafish Mao

Given that the neurochemical shifts occurred independently of gene expression changes, we hypothesized that GM-90663 acts as a direct inhibitor of Monoamine Oxidase (MAO) enzymatic activity. To test this, we combined computational modeling with in vitro biochemical assays.

**Figure 5:**
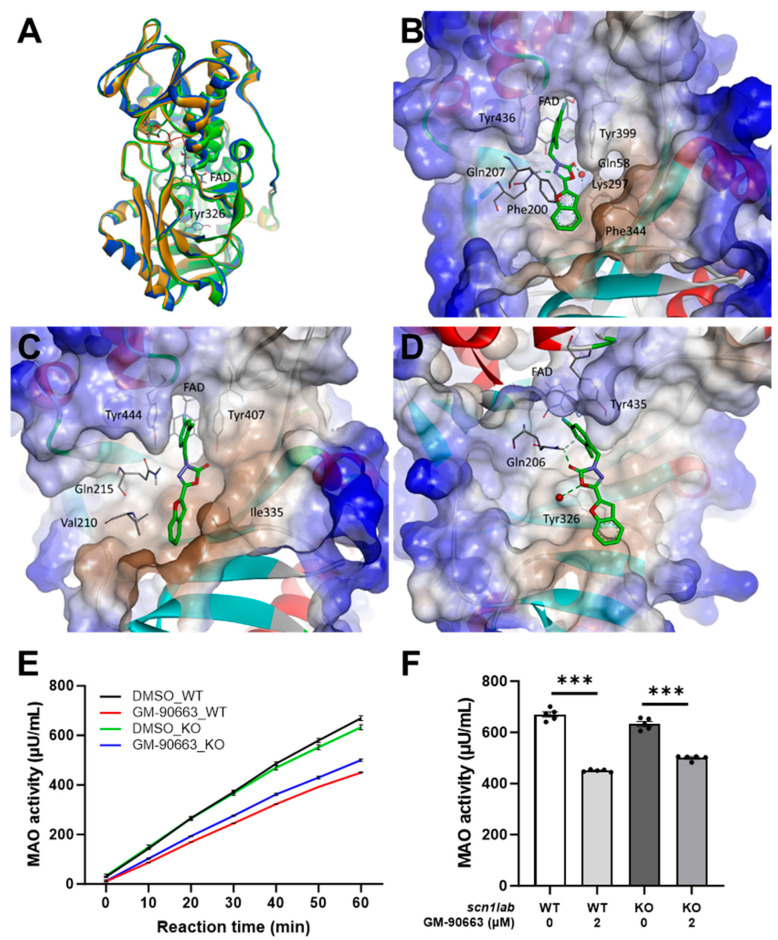
Predicted binding poses of GM-90663 in human MAO-A, MAO-B and zebrafish Mao. (**A**) Overlap of protein structures of MAO-A (yellow), MAO-B (blue), and zMao (green). (**B**–**D**) Predicted binding poses of GM-90663 in zMAO (**B**), MAO-A (**C**), and MAO-B (**D**), respectively. GM-90663 is represented in thick green sticks, and important residues and FAD in thin sticks. Hydrogen bonds are depicted in green dots, and obstructing loops are omitted for clarity. (**E**,**F**) Quantification of MAO activities by kinetic reaction time (**E**) and final reaction time (**F**) using zebrafish larvae lysates (*n* = 20). ***; *p* < 0.001.

Computational studies, including docking and molecular dynamics simulations, were carried out to identify the residues involved in the inhibition of MAOs by GM-90663. The validation of the theoretical zebrafish Mao (hereafter referred to as zMao) model was performed using the PROCHECK program, which provided satisfactory results suggesting the reliability of the model; 91.3% in the core, 8.2% allowed, 0.2–1.0% in generously allow regions, 0.2% in disallowed regions. Additionally, the structure seems to have high thermodynamic stability with an RMSD < 2.0 A in the 100 ns molecular dynamics (MD) simulation. As expected from the fact that zMao has high sequence similarity of over 70% to the human MAO-A and MAO-B, the overall structure of zMao is similar to those of the human enzymes (Figure 5A). In all three MAOs, GM-90663 showed favorable docking poses in the binding site of harmine with docking scores of −8.4, −9.5, and −7.2 kcal/mol, respectively. In zMao, the ligand binding is highly stabilized by hydrogen bonds and stacking interactions. The nitrogen atom in the oxadiazolone ring of GM-90663 forms a hydrogen bond with the side chain of Gln207. The fluoro-chlorobenzyl ring makes face-to-face stacking interactions with Tyr399 and Tyr436, and face-to-edge interaction with the isoalloxazine ring of flavin adenine dinucleotide (FAD). Additional water-mediated hydrogen bonding with Lys297 and Gly58 also contributes to the stabilization of the complex. The benzofuran ring directs to the entrance cavity space, making hydrophobic interactions with F200 and F344 (Figure 5B). In MAO-A, the ligand binding pose is quite similar to that of zMao. The oxadiazolone ring makes a hydrogen bond with Gln215 and Tyr407. The fluoro-chlorobenzyl ring is stacked between Tyr407 and Tyr444 and forms a face-to-edge interaction with FAD. The hydrophobic interaction of the benzofuran ring with Val210 and Ile335 also stabilizes the ligand binding (Figure 5C). In MAO-B, the position of GM-90663 is shifted toward helix 195–199 due to the collision with Tyr326. Ligand binding is stabilized by the hydrogen bond to Gln206, water-mediated interaction with Tyr326, and hydrophobic interactions with L171 and Y435 (Figure 5D). The major contribution to the binding of GM-90663 to MAOs comes from the hydrogen bond of the oxadiazolone ring to glutamine residues, and ring stacking interactions of the fluorochlorobenzyl ring with the tyrosine residues in the aromatic region. In MAO-B, the ligand binding pose and target specificity may be different from others due to the collision of Tyr326.

Next, we measured MAO activity using homogenates from zebrafish larvae exposed to GM-90663 to determine whether GM-90663 actually acts as an MAO inhibitor. Indeed, MAO activity was reduced in GM-90663-treated larvae regardless of wild-type and KO (Figure 5E,F). Computational studies and in vitro assays revealed that GM-90663 inhibits the enzyme activity of MAO-A and zebrafish Mao.

## 4. Discussion

Fenfluramine (FFA), which recently gained approval as an adjunctive treatment for Dravet syndrome (DS), originally demonstrated its anticonvulsant properties by influencing serotonin pathways in the *didys552* zebrafish [13,21]. Interestingly, although it was previously pulled from the market as an anti-obesity medication, FFA successfully underwent drug repurposing to be authorized for DS management [5]. In our earlier work, we established a novel zebrafish strain with a *scn1lab* loss-of-function mutation to facilitate the discovery of emerging therapeutic agents for DS. Distinct from the *didys552* point mutation, the *kri111* strain expresses a prematurely truncated form of the s*cn1lab* protein [25]. However, the morphological phenotypes of the two mutants, such as hyperpigmentation and an uninflated swim bladder, are similar. Notably, behavioral phenotypes of the two mutants are identical, since the *kri111* mutant, like the *didys552* mutant, exhibited seizure-like movements [17,25]. Therefore, we concluded that new candidate screening for DS treatment could be available using the *kri111* mutant. GM-90663 was identified as an optimized compound with improved anti-seizure efficacy in the *kri111* mutant. Although the ‘whirlpool-like’ circular behavioral pattern was not decreased, GM-90663 dose-dependently reduced hyper-activity in *scn1lab* KO. Next, we employed LC-MS/MS-based neurochemical profiling to investigate the neurological effects of GM-90663 in both wild-type and *scn1lab* KO. Our previous work has established this technology as a powerful tool for elucidating the mechanisms underlying the neuroprotective or neurotoxic effects of chemicals, utilizing various biological models, including zebrafish [34,42]. We quantitatively analyzed a range of neurotransmitters, their precursors, and metabolites, comprising six categories of the nervous system: histaminergic, cholinergic, dopaminergic, serotonergic, kynurenergic, and GABAergic, in the zebrafish model. Neurochemical profiling revealed that GM-90663 treatment increased endogenous serotonin levels in both WT and *scn1lab* KO larvae. The increase in serotonin levels induced by GM-90663 occurs independently of transcriptional changes in key serotonergic regulatory genes, such as *tph2*, *sert*, *vmat2*, and *mao*. Based on this result, we concluded that GM-90663 significantly inhibits MAO, impacting the metabolic conversion of serotonin to 5-HIAA.

Indeed, GM-90663 shows favorable docking poses in the binding sites of MAOs, with docking scores indicating strong binding affinity through docking simulation data. Especially, the ligand binding is stabilized by hydrogen bonds, stacking interactions, and hydrophobic interactions with specific residues in zMAO. In MAO-A, similar interactions are observed, with hydrogen bonds and hydrophobic interactions. Because the zMao model shares high sequence similarity (>70%) with human MAO-A and MAO-B, and its overall structure closely resembles that of human MAOs, we conclude that GM-90663 docking poses are also highly similar between human and zebrafish. MAO is an enzyme found primarily in the brain and other tissues, responsible for catalyzing the oxidation of monoamine neurotransmitters such as serotonin, dopamine, and norepinephrine. This enzyme plays a crucial role in the metabolism of neurotransmitters, regulating their levels in the brain and influencing various physiological processes, including mood, behavior, and cognition. Dysfunction of MAO has been implicated in various neurological and psychiatric disorders, making it an important target for pharmacological interventions [43]. MAO inhibitors (MAOIs) are indeed used as drugs, primarily in the treatment of depression and certain other psychiatric disorders. Examples of MAOIs include phenelzine (Nardil), tranylcypromine (Parnate), and isocarvoxazid (Marplan). These drugs work by inhibiting the activity of MAO enzymes, thereby increasing the levels of neurotransmitters such as serotonin, dopamine, and norepinephrine in the brain, which can alleviate symptoms of depression and improve mood [44]. In this study, we specifically targeted the MAO-A isoform for both computational docking and biological validation. This strategic selection was guided by the substrate preference of MAO-A, which exhibits a significantly higher affinity for 5-HT compared to MAO-B [45]. Restoration of 5-HT levels in DS model has been clinically proven to reduce seizure frequency, MAO-A inhibition represents a more direct and mechanistically relevant intervention than MAO-B inhibition.

Serotonin is a neurotransmitter primarily found in the central nervous system (CNS) and gastrointestinal tract. It plays a key role in regulating mood, appetite, sleep, and various other physiological processes. In the brain, it acts as a neurotransmitter, transmitting signals between nerve cells and modulating neural activity. Although the metabolic signature for serotonergic neurochemistry in DS is not fully understood, numerous studies have highlighted serotonergic modulation—particularly targeting serotonin receptors 1A, 1D, 2A, 2C, and 3—as a promising therapeutic strategy for this condition [19,46]. Furthermore, FFA, which is approved for DS treatment, is known for its agonistic effects on serotonin receptors 1D, 2A, and 2C [13].

Beyond its neurochemical effects on the serotonergic system, GM-90663 directly regulates neuronal excitability by modulating voltage-gated Na^+^ channels. These electrophysiological properties suggest that GM-90663 selectively suppresses high-frequency repetitive firing and reduces the probability of action potential initiation, providing a robust biophysical mechanism for its anti-seizure efficacy in Dravet syndrome models. Unlike traditional Na^+^ channel blockers, which can sometimes exacerbate seizures in DS patients due to the loss of GABAergic interneuron function, GM-90663 offers a dual-action approach. By inhibiting MAO-A, it directly increases the synaptic availability of 5-HT, a neurotransmitter significantly depleted in DS models. This serotonergic enhancement, combined with its secondary modulation of ion channels, provides a synergistic anti-seizure effect that bypasses the limitations of mono-targeted therapies.

Interestingly, while GM-90663 significantly elevated synaptic serotonin levels via MAO-A inhibition, no significant changes were observed in total GABA levels. This finding is particularly noteworthy given that Dravet syndrome is fundamentally rooted in GABAergic dysfunction. The lack of alterations in GABA concentration suggests that the anti-seizure efficacy of GM-90663 is not mediated through a direct quantitative increase in GABAergic tone, but rather through a more targeted modulation of the serotonergic system and sodium channel kinetics. This distinction is crucial, as it indicates that GM-90663 operates via an alternative neuromodulatory pathway that can bypass the refractory nature of GABA-centered treatments in DS. Furthermore, the stable GABA levels reinforce the pharmacological selectivity of our compound, demonstrating that it does not cause a non-specific surge in all inhibitory neurotransmitters, thereby potentially reducing the risk of sedative side effects often associated with GABA-enhancing drugs.

The anti-seizure efficacy and mechanism of GM-90663 observed in the *scn1lab* KO zebrafish model provide significant insights into its translational potential for human Dravet syndrome. Given the high conservation of serotonergic pathways and voltage-gated sodium channels between zebrafish and mammals, our findings are likely to be generalizable to higher vertebrate models. Furthermore, the ability of GM-90663 to target MAO enzymatic component suggests it could serve as a versatile therapeutic intervention for patients with refractory epilepsy who do not respond to conventional mono-targeted drugs. Also, regarding the pharmacological profile of GM-90663, considering potential off-target effects is essential for translational development. Our results demonstrate that the anti-seizure efficacy occurs at concentrations significantly lower than those associated with general cytotoxicity or behavioral toxicity in zebrafish larvae. While further comprehensive profiling will be necessary in future studies, the lack of adverse effects on cardiac function and early development in our model indicates a high degree of safety and selectivity for the primary targets, MAO-A and sodium channels.

Taken together, our findings demonstrate that GM-90663 exhibits an anti-seizure effect with an MAO-A inhibition for DS treatment. Additionally, whole-cell patch-clamp results revealed that GM-90663 influences the function of voltage-gated Na^+^ channels in neurons, providing insights into its potential mechanisms of action and therapeutic effects in the context of seizure treatment.

## Figures and Tables

**Table 1:** List of primer set for qRT-PCR.

*β-actin*_FP	CCGTGACATCAAGGAGAAG
*β-actin*_RP	ATACCGCAAGATTCCATACC
*c-fos*_FP	GTGAACGAAACAAGATGGCTG
*c-fos*_RP	TTTCATCCTCAAGCTGGTCAG
*mao*_FP	GCAGTCAGAGCCCGAATC
*mao*_RP	CACACCCATAAACTTGAGGAATC
*sert*_FP	TAACCACTACAGTTTGGCTTGATG
*sert*_RP	AACAGTTAACCGAGCTTGTGAT
*tph2*_FP	GCAAATACTGGGCTCGGAGA
*tph2*_RP	GAGCATGGAGGATGCAAGGT
*vmat2*_FP	TGGAGCTCTGCAGCTTTTTGTGC
*vmat2*_RP	AACGCCGGCTCCAGCATAGC

## Data Availability

The original contributions presented in this study are included in the article. Further inquiries can be directed to the corresponding authors.

## References

[1] Noebels J. (2015). Pathway-driven discovery of epilepsy genes. Nat. Neurosci..

[2] Rusina E., Simonti M., Duprat F., Cestèle S., Mantegazza M. (2023). Voltage-gated sodium channels in genetic epilepsy: Up and down of excitability. J. Neurochem..

[3] Bender A.C., Morse R.P., Scott R.C., Holmes G.L., Lenck-Santini P.-P. (2012). SCN1A mutations in Dravet syndrome: Impact of interneuron dysfunction on neural networks and cognitive outcome. Epilepsy Behav..

[4] Akiyama M., Kobayashi K., Yoshinaga H., Ohtsuka Y. (2009). A long-term follow-up study of Dravet syndrome up to adulthood. Epilepsia.

[5] Strzelczyk A., Schubert-Bast S. (2022). A Practical Guide to the Treatment of Dravet Syndrome with Anti-Seizure Medication. CNS Drugs.

[6] Frampton J.E. (2023). Fenfluramine: A Review in Dravet and Lennox-Gastaut Syndromes. Drugs.

[7] Nickels K.C., Wirrell E.C. (2017). Stiripentol in the management of epilepsy. CNS Drugs.

[8] Fisher J.L. (2011). Interactions between modulators of the GABAA receptor: Stiripentol and benzodiazepines. Eur. J. Pharmacol..

[9] Ryan M. (2020). Cannabidiol in epilepsy: The indications and beyond. Ment. Health Clin..

[10] Schoonjans A.-S., Ceulemans B. (2019). An Old Drug for a New Indication: Repurposing Fenfluramine From an Anorexigen to an Antiepileptic Drug. Clin. Pharmacol. Ther..

[11] Schoonjans A.-S., Ceulemans B. (2022). A critical evaluation of fenfluramine hydrochloride for the treatment of Dravet syndrome. Expert Rev. Neurother..

[12] Martin P., De Witte P.A.M., Maurice T., Gammaitoni A., Farfel G., Galer B. (2020). Fenfluramine acts as a positive modulator of sigma-1 receptors. Epilepsy Behav..

[13] Sourbron J., Smolers I., de Witte P.A.M., Lagae L. (2017). Pharmacological Analysis of the Anti-epileptic Mechanisms of Fenfluramine in scn1a Mutant Zebrafish. Front. Pharmacol..

[14] Best J. (2008). Zebrafish: An in vivo model for the study of neurological diseases. Neuropsychiatr. Dis. Treat..

[15] Zon L.I., Peterson R.T. (2005). In vivo drug discovery in the zebrafish. Nat. Rev. Drug Discov..

[16] Howe K., Clark M.D., Torroja C.F., Torrance J., Berthelot C., Muffato M., Collins J.E., Humphray S., McLaren K., Matthews L. (2013). The zebrafish reference genome sequence and its relationship to the human genome. Nature.

[17] Baraban S.C., Dinday M.T., Hortopan G.A. (2013). Drug screening in Scn1a zebrafish mutant identifies clemizole as a potential Dravet syndrome treatment. Nat. Commun..

[18] Dinday M.T., Baraban S.C. (2015). Large-Scale Phenotype-Based Antiepileptic Drug Screening in a Zebrafish Model of Dravet Syndrome. eNeuro.

[19] Griffin A., Hamling K.R., Knupp K., Hong S., Lee L.P., Baraban S.C. (2017). Clemizole and modulators of serotonin signalling suppress seizures in Dravet syndrome. Brain.

[20] Grone B.P., Qu T., Baraban S.C. (2017). Behavioral Comorbidities and Drug Treatments in a Zebrafish *scn1lab* Model of Dravet Syndrome. eNeuro.

[21] Sourbron J., Partoens M., Scheldeman C., Zhang Y., Lagae L., Witte P. (2019). Drug repurposing for Dravet syndrome in *scn1Lab*^−/−^ mutant zebrafish. Epilepsia.

[22] Griffin A., Anvar M., Hamling K.R., Baraban S.C. (2020). Phenotype-Based Screening of Synthetic Cannabinoids in a Dravet Syndrome Zebrafish Model. Front. Pharmacol..

[23] Muto A., Orger M.B., Wehman A.M., Smear M.C., Kay J.N., Page-Mccaw P.S., Gahtan E., Xiao T., Nevin L.M., Gosse N.J. (2005). Forward Genetic Analysis of Visual Behavior in Zebrafish. PLoS Genet..

[24] Schoonheim P.J., Arrenberg A.B., Del Bene F., Baier H. (2010). Optogenetic localization and genetic perturbation of saccade-generating neurons in zebrafish. J. Neurosci..

[25] Kim D.G., Hwang K.-S., Ahn S.H.A., Kim S.S., Son Y., Park S.B., Jung W.H., Shin D.-S., Cho S.H., Choi B.W. (2026). Development of Novel Small-Molecule Targeting SCN1A-Associated Severe Myoclonic Epilepsy of Infancy. J. Med. Chem..

[26] Westerfield M. (2000). The Zebrafish Book. A Guide for the Laboratory Use of Zebrafish (Danio rerio).

[27] Hwang K.-S., Kan H., Kim S.S., Chae J.S., Yang J.Y., Shin D.-S., Ahn S.H., Ahn J.H., Cho J.-H., Jang I.-S. (2020). Efficacy and pharmacokinetics evaluation of 4-(2-chloro-4-fluorobenzyl)-3-(2-thienyl)-1,2,4-oxadiazol-5(4H)-one (GM-90432) as an anti-seizure agent. Neurochem. Int..

[28] Hwang K.-S., Son Y., Kim S.S., Shin D.-S., Lim S.H., Yang J.Y., Jeong H.N., Lee B.H., Bae M.A. (2022). Size-Dependent Effects of Polystyrene Nanoparticles (PS-NPs) on Behaviors and Endogenous Neurochemicals in Zebrafish Larvae. Int. J. Mol. Sci..

[29] Akaike N., Moorhouse A.J. (2003). Techniques: Applications of the nerve–bouton preparation in neuropharmacology. Trends Pharmacol. Sci..

[30] Jang I.-S., Nakamura M., Ito Y., Akaike N. (2006). Presynaptic GABA_A_ receptors facilitate spontaneous glutamate release from presynaptic terminals on mechanically dissociated rat CA3 pyramidal neurons. Neuroscience.

[31] Hamill O.P., Marty A., Neher E., Sakmann B., Sigworth F.J. (1981). Improved patch-clamp techniques for high-resolution current recording from cells and cell-free membrane patches. Pflügers Arch..

[32] Murase K., Ryu P.D., Randic M. (1989). Excitatory and inhibitory amino acids and peptide-induced responses in acutely isolated rat spinal dorsal horn neurons. Neurosci. Lett..

[33] Oikonomou G., Altermatt M., Zhang R.-w., Coughlin G.M., Montz C., Gradinaru V., Prober D.A. (2019). The Serotonergic Raphe Promote Sleep in Zebrafish and Mice. Neuron.

[34] Kim S.S., Lee H.-Y., Song J.S., Bae M.A., Ahn S. (2021). UPLC-MS/MS-based profiling of 31 neurochemicals in the mouse brain after treatment with the antidepressant nefazodone. Microchem. J..

[35] Schrödinger L. (2021). Schrödinger Release 2021–3: Maestro.

[36] Holmes G.L. (2015). Cognitive impairment in epilepsy: The role of network abnormalities. Epileptic Disord..

[37] Park J.-S., Ryu J.-H., Choi T.-I., Bae Y.-K., Lee S., Kang H.J., Kim C.-H. (2016). Innate Color Preference of Zebrafish and Its Use in Behavioral Analyses. Mol. Cells.

[38] Randlett O., Wee C.L., Naumann E.A., Nnaemeka O., Schoppik D., Fitzgerald J.E., Portugues R., Lacoste A.M.B., Riegler C., Engert F. (2015). Whole-brain activity mapping onto a zebrafish brain atlas. Nat. Methods.

[39] Cruz-Mendoza F., Jauregui-Huerta F., Aguilar-Delgadillo A., García-Estrada J., Luquin S. (2022). Immediate Early Gene c-fos in the Brain: Focus on Glial Cells. Brain Sci..

[40] Baraban S.C., Taylor M.R., Castro P.A., Baier H. (2005). Pentylenetetrazole induced changes in zebrafish behavior, neural activity and c-fos expression. Neuroscience.

[41] Catterall W.A. (2014). Sodium Channels, Inherited Epilepsy, and Antiepileptic Drugs. Annu. Rev. Pharmacol. Toxicol..

[42] Kim S.S., Hwang K.-S., Yang J.Y., Chae J.S., Kim G.R., Kan H., Jung M.H., Lee H.-Y., Song J.S., Ahn S. (2020). Neurochemical and behavioral analysis by acute exposure to bisphenol A in zebrafish larvae model. Chemosphere.

[43] Bortolato M., Shih J.C. (2011). Behavioral outcomes of monoamine oxidase deficiency: Preclinical and clinical evidence. Int. Rev. Neurobiol..

[44] Stahl S.M., Felker A. (2008). Monoamine oxidase inhibitors: A modern guide to an unrequited class of antidepressants. CNS Spectr..

[45] Bortolato M., Chen K., Shih J.C. (2008). Monoamine oxidase inactivation: From pathophysiology to therapeutics. Adv. Drug Deliv. Rev..

[46] Sourbron J., Lagae L. (2022). Serotonin receptors in epilepsy: Novel treatment targets?. Epilepsia Open.

